# A Nanostructured Lipid System to Improve the Oral Bioavailability of Ruthenium(II) Complexes for the Treatment of Infections Caused by *Mycobacterium tuberculosis*

**DOI:** 10.3389/fmicb.2018.02930

**Published:** 2018-12-06

**Authors:** Patricia B. da Silva, Eduardo Sinésio de Freitas, Mariana Cristina Solcia, Paula Carolina de Souza, Monize Martins da Silva, Alzir Azevedo Batista, Carlos E. Eismann, Ana Marta C. M. Rolisola, Amauri A. Menegário, Rosilene Fressatti Cardoso, Marlus Chorilli, Fernando R. Pavan

**Affiliations:** ^1^School of Pharmaceutical Sciences, Universidade Estadual Paulista, Araraquara, Brazil; ^2^Department of Chemistry, Universidade Federal de São Carlos, São Carlos, Brazil; ^3^Environmental Studies Center, Universidade Estadual Paulista, Rio Claro, Brazil; ^4^Department of Clinical Analysis and Biomedicine, State University of Maringa, Maringá, Brazil

**Keywords:** ruthenium(II) complexes, tuberculosis, nanotechnology, oral bioavailability, ICP-MS

## Abstract

Tuberculosis (TB) is an infectious, airborne disease caused by the bacterium *Mycobacterium tuberculosis* that mainly affects the lungs. Fortunately, tuberculosis is a curable disease, and in recent years, death rates for this disease have decreased. However, the existence of antibiotic-resistant strains and the occurrence of co-infections with human immunodeficiency virus (HIV), have led to increased mortality in recent years. Another area of concern is that one-third of the world′s population is currently infected with *M. tuberculosis* in its latent state, serving as a potential reservoir for active TB. In an effort to address the failure of current TB drugs, greater attention is being given to the importance of bioinorganic chemistry as an ally in new research into the development of anti-TB drugs. Ruthenium (Ru) is a chemical element that can mimic iron (Fe) in the body. In previous studies involving the following heteroleptic Ru complexes, [Ru(pic)(dppb)(bipy)]PF_6_ (SCAR1), [Ru(pic)(dppb)(Me-bipy)]PF_6_ (SCAR2), [Ru(pic)(dppb)(phen)]PF_6_ (SCAR4), *cis*-[Ru(pic)(dppe)_2_]PF_6_ (SCAR5), and [Ru(pic)(dppe)(phen)]PF_6_ (SCAR7), we observed excellent anti-TB activity, moderate cell-toxicity, and a lack of oral bioavailability in an *in vivo* model of these complexes. Therefore, the objective of this study was to evaluate the toxicity and oral bioavailability of these complexes by loading them into a nanostructured lipid system. The nanostructured lipid system was generated using different ratios of surfactant (soybean phosphatidylcholine, Eumulgin^®^, and sodium oleate), aqueous phase (phosphate buffer with a concentration of 1X and pH 7.4), and oil (cholesterol) to generate a system for the incorporation of Ru(II) compounds. The anti-TB activity of the compounds was determined using a microdilution assay with Resazurin (REMA) against strains of *M. tuberculosi*s H_37_Rv and clinical isolates resistant. Cytotoxicity assay using J774.A1 cells (ATCC TIB-67) and intra-macrophage activity were performed. The oral bioavailability assay was used to analyze blood collected from female BALB/C mice. Plasma collected from the same mice was analyzed via inductively coupled plasma mass spectrometry (ICP-MS) to quantify the number of Ru ions. The complexes loaded into the nanostructured lipid system maintained *in vitro* activity and toxicity was found to be reduced compared with the compounds that were not loaded. The complexes showed intra-macrophagic activity and were orally bioavailable.

## Introduction

*Mycobacterium tuberculosis* is the main causative agent of tuberculosis (TB). TB causes the highest number of deaths worldwide. In 2016, TB caused 1.3 million deaths and 6.3 million new cases of TB were reported worldwide. The standard treatment for TB currently recommended by the World Health Organization (WHO) for susceptible infections is the concomitant use of the following four antibiotics: rifampicin (RFP), ethambutol (ETH), pyrazinamide (PZA), and isoniazid (INH) administered as a single dose daily for 6 months. This therapy, which is known as first-line therapy, can be successful as long as it is maintained for the full 6-month period. This current therapy still long, when it succeeds (World Health Organization [WHO], 2017a).

The long treatment duration together with a variety of associated adverse events, particularly hepatotoxicity (a potential side-effect of all of the antibiotics included in the treatment), often results in the therapy being discontinued by the patient before the end of the 6-month period (Senousy et al., 2010). Discontinuation of therapy can lead to the emergence of resistant strains and therefore treatment of infections caused by these strains will depend on the sensitivity test, and the treatment may take 2 years. Four different types of resistance to anti-tuberculosis drugs have been described in the current literature. Mono-resistance is the ability of an *M. tuberculosis* strain to resist only one of the four drugs included in first-line therapy. In multi-drug-resistance (MDR-TB), *M. tuberculosis* has resistance to both INH and RMP. In XDR-TB resistance, *M. tuberculosis* is able to resist a fluoroquinolone (moxifloxacin, gatifloxacin, or ofloxacin), in addition to INH and RMP. Some strains are also known to be resistant to injectable antimicrobial agents (aminoglycosides) and a fully resistant (TDR-TB) strain has been reported in some countries (Italy, India and Iran) as being resistant to all antimicrobials currently being used to treat TB. However, the existence of this strain has not yet been officially recognized by the WHO (Mali and Morbale, 2016; World Health Organization [WHO], 2017a).

To address the lack of effective antimicrobials to treat TB and the current drawbacks of first-line therapy, the WHO encourages research into the development of new antimicrobials for TB and recommends that the new drugs should have the following characteristics: (1) reduction of the treatment period, (2) activity against resistant strains, (3) activity against non-replicating bacteria, (4) an intra-macrophagic effect, (5) good bioavailability and few adverse events, and (6) be affordable to patients globally (Bhat et al., 2017).

The development of new molecules effective against *M. tuberculosis* has involved all areas of medicinal chemistry. However, in recent years, there has been a steady increase in the number of studies being conducted in the area of bio-inorganic chemistry. These studies aim to develop new anti-mycobacterial compounds that contain metals/metal ions (Viganor et al., 2015). Ruthenium (Ru) is a chemical element that has the ability to mimic iron (Fe) in the human body. It has low toxicity and is able to form stable complexes (Allardyce and Dyson, 2001).

Numerous Ru(II) complexes exhibiting anti-mycobacterial activity against *M. tuberculosis* have been described in the literature. Do Nascimento et al. (2008) synthesized, characterized, and evaluated the anti-*M. tuberculosis* activity of four different Ru(II) complexes containing phosphine and diimine and the ligand HSpymMe_2_, 4,6-dimethyl-2-mercaptopyrimidine. [Ru(SpymMe_2_)(dppb)(N–N)]PF_6_, N–N = bipy (1) and Me-bipy (2), bipy = 2,2′-bipyridine and Me-bipy = 4,4′-dimethyl-2,2′-bipyridine were cationic complexes. The other complexes were *cis*-[RuCl_2_(dppb)(N–N)], N–N = bipy (3), and Me-bipy (4). The minimum inhibitory concentration (MIC) of Ru complexes 1–4 was 0.78, 0.78, 3.9, and 6.25 μg mL^-1^, respectively. The results demonstrate that the compounds exhibit good anti-mycobacterial activity.

A study by Pavan et al. (2010b) describes the synthesis, characterization, and determination of the MIC against *M. tuberculosis* H_37_Rv of three ruthenium(II) phosphine/picolinate, *cis*-[Ru(pic)(dppm)_2_] PF_6_ (1), *cis*-[Ru(pic)(dppe)_2_]PF_6_ (2), and [Ru(pic)_2_ (PPh_3_)_2_] (3) [pic = 2-pyridinecarboxylate; dppm = bis(diphenylphosphino)methane; dppe = 1,2-bis(diphenylphosphino)ethane; PPh_3_ = triphenylphosphine]. MIC values for the complexes 1–3 were 0.78, 0.256, and >50 μg ml^-1^ respectively. These results indicate that these complexes show promise as anti-mycobacterial agents as the data are comparable to those of second-line drugs currently used to treat TB (Collins and Franzblau, 1997).

Ru(II) complexes with hydroxypyridinecarboxylates as ligand: [Ru(2-OHnic)(dppb)(bipy)]PF_6_ (1), [Ru(6-OHnic) (dppb)(bipy)]PF_6_ (2) and [Ru(3-OHpic)(dppb)(bipy)]PF_6_ (3) [dppb = 1,4 bis(diphenylphosphino)butane, bipy = 2,2′-bipyridine, 2-OHnicH = 2-hydroxynicotinic acid, 6-OHnicH = 6-hydroxynicotinic acid and 3-OHpicH = 3-hydroxypicolinic acid] were evaluated for activity against *M. tuberculosis* H_37_Rv ATCC 27294 and cytotoxicity in the VERO CCL-81 cell line. The complexes 1-3 exhibited MIC values of 0.39, 6.3, and 3.1 μg ml^-1^ respectively. They showed no cytotoxicity and therefore had a high selectivity index (SI = IC_50_/MIC) (Barbosa et al., 2015).

A study by Aguiar et al. (2015) demonstrated the anti-tubercular activity of Ru(II) INH complexes: *trans*-[Ru(NH_3_)_4_ (SO_2_)(ina)](BF4)_2_ (1), *trans*-[Ru(NH_3_)_4_(SO_2_)(INH)](BF_4_)_2_ (2) [ina = isonicotinic acid, INH = isoniazid], which had MIC values of > 25 and 0.88 μg mL^-1^, respectively.

*In vitro* activities of Ru(II) phosphine/diimine/picolinate complexes ([Ru(pic)(dppb)(bipy)]PF_6_ (SCAR1), [Ru(pic)(dppb) (Me-bipy)]PF_6_ (SCAR2), [Ru(pic)(dppb)(phen)]PF_6_ (SCAR4), cis-[Ru(pic)(dppe)_2_]PF_6_ (SCAR5), *cis*-[RuCl_2_(dppb)(bipy)] (SCAR6) and [Ru(pic)(dppe)(phen)]PF_6_ (SCAR7) [pic = 2-pyridinecarboxylic acid anion, dppb = 1,4-bis(diphenylphosphino)butane, bipy = bipyridine, Me-bipy = = 4,4′-dimethyl-2,2′-bipyridine, phen = 1,10-phenanthroline, dppe = 1,2-bis(diphenylphosphino)ethane]) against *M. tuberculosis* were studied by Pavan et al. (2013). All of the SCAR compounds exhibited anti-*M. tuberculosis* activity against both susceptible and drug-resistant strains as well as non-replicating, persistent bacteria and showed no toxicity or mutagenicity.

The low solubility of metallic compounds in water makes it impossible to study their biological activity in animals. Therefore, alternatives have been sought for the delivery of metallic compounds using specialized methods that effectively compartmentalize various groups of molecules and modify their behavior in the organism (Sato et al., 2015).

The nanostructured lipid system (NLS) as a microemulsion (ME) is a system that was developed by Hoar and Schulman in 1943. The system is thermodynamically stable and isotropically translucent, and is composed of two immiscible liquids (oil/water) stabilized by an interfacial film of surfactants. The number of pharmaceuticals utilizing novel drug delivery systems has increased significantly in recent years and is expected to increase further (Dos Santos Ramos et al., 2018). There continues to be a strong focus on the design and development of new drug-delivery systems to increase the effectiveness of existing drugs. Recently, a large number of studies have focused on the use of MEs as drug-delivery systems (Ho et al., 1996; Lawrence, 1996), particularly after the discovery of a successful ME for oral use, called Neoral^®^ (Lawrence, 1996). NLSs have a low surface tension. This is an important property that improves the solubility, stability, and bioavailability of molecules loaded into this carrier and thereby enhances absorption and permeability (Dos Santos Ramos et al., 2018).

There are several reports on the methods for enhancing the solubility, and hence the bioavailability, of molecules. Ghosh et al. (2006) developed an NLS, which consisted of non-ionic Labrasol at 32% (as surfactant), Plurol Oleique at 8% (as cosurfactant), Labrafac at 10%, and water at 50%, with the aim of improving the bioavailability of acyclovir, a poorly soluble drug. The results of an *in vivo* study showed that the bioavailability of acyclovir improved 12.78 times when it loaded into the NLS following oral administration, compared to that of commercially available acyclovir tablets.

In a study conducted by Hu et al. (2011), *in vivo* experiments in rats were performed to evaluate the oral bioavailability of ibuprofen loaded into a lipid formulation composed of 17% Labrafl M 1944CS, 28% Cremophor RH40/Transcutol P (3:1, w/w), and 55% water and granule formulation. The authors observed that the lipid system rendered the drug, ibuprofen, 1.9 times more bioavailable than its granule formulation.

Curcumin has several pharmacological activities. However, it has low solubility in water. In order to improve the oral bioavailability of the drug, Hu et al. (2012) developed a lipid system containing an oil phase composed of Capyrol 90, with Cremophor RH40 as the surfactant and as a Transcutol P aqueous solution as the co-surfactant. A study in rats using the curcumin-containing formulation showed an improvement in the solubility, and consequently the bioavailability of the drug, which was 22.6 times greater than that of the suspension.

In order to improve the oral bioavailability of felodipine, Singh et al. (2018) developed an NLS consisting of α-linolenic acid (oil phase), Tween 80 (surfactant), and isopropanol (co-surfactant). The authors observed an improvement of 308.3% in the bioavailability of the developed formulation compared to that of the commercial formulation.

The purpose of this study was to analyze the following five loaded Ru(II) compounds: [Ru(pic)(dppb)(bipy)]PF_6_ (SCAR1); [Ru(pic)(dppb)(Me-bipy)]PF_6_ (SCAR2); [Ru(pic) (dppb)(phen)]PF_6_ (SCAR4); *cis*-[Ru(pic)(dppe)_2_]PF_6_ (SCAR5) and [Ru(pic)(dppe)(phen)]PF_6_ (SCAR7), [pic = 2-pyridinecarboxylic acid anion, dppb = 1,4-bis(diphenylphosphino)butane, bipy = bipyridine, Me-bipy = =4,4′-dimethyl-2,2′-bipyridine, phen = 1,10-phenanthroline, dppe = 1,2-bis(diphenylphosphino)ethane]) into the NLS composed of a 10% oil phase (i.e., cholesterol), 10% surfactant (i.e., soy phosphatidylcholine, sodium oleate, and Eumulgin^®^ HRE 40 [castor oil polyoxyl-40-hydrogenated] in a proportion of 3:6:8), and an 80% aqueous phase (i.e., phosphate buffer with a concentration of 1X and pH 7.4) as a strategy to enhance the oral bioavailability. The cytotoxicity of these complexes was evaluated in mouse macrophages (J774A.1 ATCCb TIB-67). Intramacrophage activity was determined using an *in vitro* infection model using the *M. tuberculosis* strain H_37_Rv (ATCC 27294), and *in vivo* oral bioavailability was analyzed using a rapid *screening* method.

## Materials and Methods

### Inorganic Compounds

**FIGURE 1:**
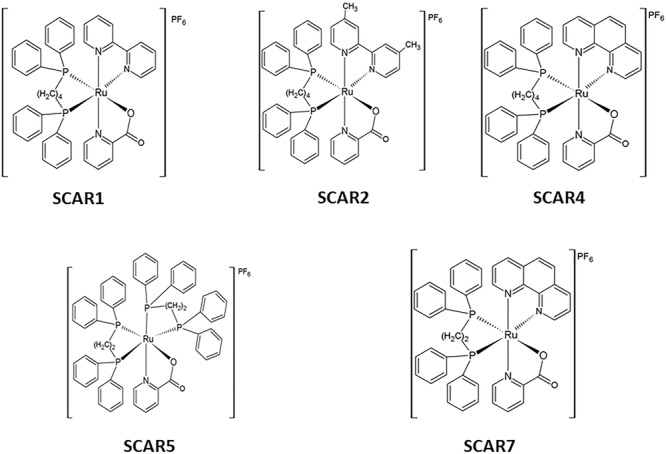
Structures of the SCARs complexes.

The complexes [Ru(pic)(dppb)(bipy)]PF_6_ (SCAR1), [Ru(pic)(dppb)(Me-bipy)]PF_6_ (SCAR2), [Ru(pic)(dppb)(phen)] PF_6_ (SCAR4), cis-[Ru(pic)(dppe)_2_]PF_6_ (SCAR5), and [Ru(pic)(dppe)(phen)]PF_6_ (SCAR7) were synthesized according to the methodology previously described by our group (Pavan et al., 2011). The structures of the SCARs complexes are shown in Figure 1.

### Nanostructured Lipid System (NLS)

#### NLS Preparation

The NLS was synthesized as previously described by our group (De Freitas et al., 2014; da Silva et al., 2016) and had the following composition: 10% oil phase (cholesterol), 10% surfactant (a mixture of soy phosphatidylcholine, sodium oleate, and Eumulgin HRE 40 [polyoxyl 40 castor oil-hydrogenated]; 3:6:8), and 80% aqueous phase (phosphate buffer with a concentration of 1X and pH 7.4). The NLS was prepared by the sonication method using rod sonicator with 700 watts of power and 20Hz of frequency (Q700 of QSonica^®^, Newtown, CT, United States). The mixture was sonicated for 10 cycles of 1 min with 15% amplitude and 30 s intervals between then. The flask containing the mixture was maintained in ice bath throughout the process. At the end of 10 cycles, the NLSs were centrifuged for removing the titanium residues released during the sonication process; The NLSs were synthesized in the previous day (24 h) before of the assays.

#### Preparation of the SCARs-Loaded Into the NLS

The Ru compounds studied were incorporated as follows:

For *in vitro* experiments, a mass of 5 mg of the respective compound of interest was weighed and solubilized in 1 mL NLS. The solution obtained was sonicated on the rod sonicator with 700 watts of power and 20 Hz of frequency (Q700 of QSonica^®^, Newtown, CT, United States) for 3 min in a 15% amplitude ice bath for complete incorporation. The sample was then centrifuged to remove the titanium residues from the sonication process and the concentration obtained was 5 mg ml^-1^.

For the *in vivo* experiments, the same methodology of incorporation was performed, however, 10 mg of the respective compound of ruthenium(II) was used for 1 ml NLS, consequently the concentration obtained in the sample was 10 mg ml^-1^, which corresponds to a dose of 300 mg/kg body weight.

#### NLS Characterization: Mean Hydrodynamic Diameter, Polydispersity Index (PDI) and Zeta Potential (ZP)

##### Determination of the mean hydrodynamic diameter and polydispersity index (PDI)

The mean hydrodynamic diameter (Z-ave or d.nm) and polydispersity index (PDI) were determined by dynamic light scattering (Malvern Instruments, San Diego, CA, United States). Each formulation of NLS was diluted (100 μL sample in 900 μL ultrapure water). All experiments were conducted in triplicate at 25°C and the results were expressed as mean ± standard deviation (mean ± SD).

##### Zeta potential analysis

The zeta potential of the NLSs was assessed by the electrophoretic mobility of the particles according to the Helmholtz–Smoluchowski equation, and processed using the Zetasizer Nano equipment software NS (Malvern Instruments, San Diego, CA, United States). Each formulation of NLSs was diluted in ultrapure water at a ratio of 1:100. The ZP analysis was performed in triplicate at 25°C and the results were expressed as mean ± S.D.

### Biological Assays

#### Determination of *in vitro* Activity Against *M. tuberculosis* H_37_Rv and Resistant Clinical Isolates

The anti-*M. tuberculosis* activity of the compounds was determined using the Resazurin Microtiter Assay (REMA) method according to Palomino et al. (2002). Stock solutions of the tested compounds were prepared in dimethyl sulfoxide (DMSO), incorporated into the NLS, and diluted in Middlebrook 7H9 broth (Difco, Detroit, MI, United States) supplemented with oleic acid, albumin, dextrose, and catalase (OADC enrichment - BBL/Becton-Dickinson, Detroit, MI, United States) to obtain a final drug concentration range of 0.09–25 μg ml^-1^. A suspension of either the *M. tuberculosis* H_37_Rv ATCC 27294 or the clinical isolate was cultured in Middlebrook 7H9 broth supplemented with OADC and 0.05% Tween 80. The culture was frozen at -80°C in aliquots. Two days later, the CFU/ml of the aliquot was determined. The concentration was adjusted to 5 × 10^5^ CFU/ml, and 100 μl of the inoculum was added to each well of a 96-well microplate together with 100 μl of the compounds. The samples were set up in triplicate. The plate was incubated for 7 days at 37°C. Next, 30 μl of 0.01% resazurin (solubilized in water) was added to each of the wells of the plate. The fluorescence of the wells was read after 24 h of incubation using a Cytation 3 (Biotek^®^, Winooski, VT, United States). The MIC_90_ was defined as the lowest concentration resulting in 90% growth inhibition of *M. tuberculosis*.

#### *In vitro* Cytotoxic Activity

The cytotoxicity of the complexes solubilized in DMSO and loaded into the NLS was measured in mouse macrophages (J774A.1 ATCCb TIB-67) as described by Pavan et al. (2010a). The cells were incubated at 37°C with 5% CO_2_ on plates with a surface area of 12.50 cm^2^ in 10 ml RPMI (Vitrocell^®^, Campinas, SP, Brazil) supplemented with 10% fetal bovine serum, gentamicin sulfate (50 mg l^-1^) and amphotericin B (2 mg l^-1^). This technique consists of collecting the cells using a scraper, centrifuging (2000 rpm for 5 min), counting the number of cells in a Newbauer chamber, and then adjusting the concentration to 3.4 × 10^5^ cells ml^-1^ in RPMI. Next, 200 μl suspension was deposited into each well of a 96-well microplate and incubated at 37°C in an atmosphere of 5% CO_2_ for 24 h to allow the cells to attach to the plate. Dilutions of the test compounds were prepared to obtain concentrations from 500 to 1.95 μg ml^-1^. The dilutions were added to the cells after the removal of the medium and any cells that did not adhere, and the plate was then incubated again for 24 h. The cytotoxicity of the compounds was determined by adding 30 μl developer of resazurin and read after a 6-h incubation. The reading was performed in a microplate Cytation 3 (Biotek^®^, Winooski, VT, United States) reader using excitation and emission filters at wavelengths of 530 and 590 nm, respectively. The cytotoxicity (IC_50_) was defined as the highest concentration of compound inhibiting the growth of at least 50% of the cells and was obtained from non-linear regression using GraphPad Prism 7.0 for Windows.

#### Intra-macrophage Activity

Intra-macrophage activity was determined in an *in vitro* infection model using the *M. tuberculosis* H_37_Rv (ATCC 27294) strain. The suspension was seeded onto Middlebrook 7H10 (solid medium), supplemented with OADC for CFU/ml counts. Cultures of J774A.1 (ATCC TIB–67) murine macrophage-like cell line were cultured with RPMI 1640 complete medium (10% BFS) at 37°C and 5% CO_2_. Once the cells reached confluency, they were detached and counted. For analysis of intra-macrophage activity, 5 × 10^5^ cells/ml were seeded into each well of a 24-well plate (TPP^®^, 1 ml per well). The plate was then incubated at 37°C under a 5% CO_2_ atmosphere for 24 h to allow cell-adhesion. Bacterial suspensions of *M. tuberculosis* were adjusted and diluted in RPMI 1640 complete medium. The cells were infected by incubating them with two bacteria per cell for 2 h at 37°C. Following infection, amikacin suspension in RPMI medium was added to each well at a final concentration of 200 μg/ml and the plates were incubated for a further 2 h to eliminate bacteria that were not phagocytosed. Next, each well was washed x3 with PBS with a concentration of 1X and pH 7.4. The total number of cell-associated mycobacteria was initially determined in the following manner: J774A.1 cells were lysed in 1 ml of sterile distilled water containing 0.1% Triton X–100 per well to confirm the infection (T0h). All of the compounds in either DMSO or in micro-emulsion were diluted in RPMI 1640 complete medium at a range of concentrations determined in previous cytotoxicity assays (1 ml per well at each concentration). Following this, the plates were incubated for 72 h at 37°C and 5% CO_2,_ and then washed and lysed with 1 ml of sterile distilled water containing 0.1% Triton X–100 per well. The CFU was determined and the percent inhibition by each compound at each concentration was calculated after comparison with the positive control. As a standard test, intracellular inhibition by RFP was determined. Each test was set up in triplicate (Dos Santos Fernandes et al., 2017).

#### *In vivo* Oral Bioavailability Assay

##### Plasma collection for evaluation of oral bioavailability, through pharmacokinetic *screening* in BALB/c mice

Female, 4–8-weeks old BALB/c mice weighing 15–20 g were purchased from the central animal facilities of Campinas State University (UNICAMP, Campinas, SP, Brazil). The protocol for plasma collection was approved by the Ethics Committee on the Use of Animals in Research – CEUA School of Pharmaceutical Sciences of Araraquara – UNESP (Protocol number 24/2013). The mice were maintained in polycarbonate cages at 23 ± 2°C, 56 ± 2% humidity, in a 12 h light/dark cycle, under specific pathogen-free conditions (in a positive-pressure cabinet), and the animals were provided with food and water *ad libitum*. The *in vivo* oral bioavailability of the SCAR complexes, either solubilized in sunflower oil or loaded into the NLS, was determined according to a previously reported method (Gruppo et al., 2006). The animals (*n* = 4 animals/group) received a dose of 300 mg/kg body weight via oral gavage which corresponds to a concentration 10 mg ml^-1^ of the complex. The groups studied were SCAR1, SCAR2, SCAR4 and SCAR7 solubilized in sunflower, SCAR1-loaded, SCAR2-loaded, SCAR4-loaded, SCAR5-loaded and SCAR7-loaded into the NLS, the control groups were sunflower and NLS, and negative control that received water. At 20 min, 1, 2, and 4 h following drug administration, blood was drawn from two mice via the retro-orbital vein, draining the sub-mandibular vein and the face of the mice at the point where the origins of the jugular vein join, to allow the collection of drops of blood exuding from the point of penetration (Golde et al., 2005). The plasma was then separated from the blood and stored at -70°C for quantification.

##### Specimen preparation for inductively coupled plasma mass spectrometry (ICP-MS)

The Ru(II) complexes were quantified using Inductively Coupled Plasma Mass Spectrometry (ICP-MS) X-Series 2 (Thermo Scientific^®^, Germany) (Batista et al., 2009). Fifty microliters of plasma samples and 5950 μL of bi-distilled nitric acid 20% (v/v) were digested in a microwave (Berghof speedwave D 72800), at 170°C. Following digestion, 2 ml of the samples were transferred to a 15 ml conical tube and 800 μl of the internal standard stock solution (Bi, Ho, In, Li-6, Sc, Tb, Y) was added in order to obtain a final concentration of internal standard in the samples of 100 μg L^-1^ and another 5.2 ml of milli-Q water, leaving the acidity of the solution at 5% m/v. For the calibration, mono elemental solution of Ru was used at the following concentrations: 25, 50, 100, 250, 500, and 1000 ppt. Diluent solution was used as the blank. The solutions were used to construct an analytical curve. In all solutions, the internal standard was also added in order to correct signal fluctuations (counts per minute). During the experiment, the method of recovery validation test was performed and gave a result of 95–105%.

The detection limit (DL) and the quantification limit (QL) of the method were determined by Martincic et al. (2014), by reading the blank of the curve with ten replicates, and by applying the following equations:

(1) $$\begin{array}{c} \mathrm{DL}\;=\;3\;\times \;(Standard deviation of white)\mu gL^{-}{}^{1} \end{array}$$

(2) $$\begin{array}{c} \mathrm{LQ}\;=\;10\;\times \;(Standard deviation of white)\mu gL^{-}{}^{1} \end{array}$$

The *in vivo* oral bioavailability was determined as the mean of two independent assays.

## Results and Discussion

Some data presented in this paper belong to a master′s dissertation by Eduardo Sinésio de Freitas (Freitas, 2015).

### NLS Characterization

Table 1 shows the mean values and standard deviation of particle sizes, PDI and ZP for the NLS and for the coordination compounds loaded into the nanosystem. Looking at the table, the particle size of the NLS was 171.6 ± 0.8 nm. The incorporation of the SCARs compounds induced a small variation in particle size, without exception, ranging from 185.1 ± 0.6 to 213.0 ± 2.5 nm. All values obtained are in the range of 10–200 nm (100–2000 Å), ideal range for NLS (Formariz et al., 2005). When comparing the NLS and the formulations containing the SCARs-loaded into the nanosystem, there was a small increase in the size of the particle diameter, a strong indication that the SCARs were incorporated into the NLS.

**Table 1:** Values expressed as mean ± standard deviation of the mean hydrodynamic diameter, polydispersity index (PDI) and zeta potential of the NLS and formulations containing the SCARs complexes loaded into the NLS (*n* = 3).

Formulation	Mean Diameter ± SD (nm) ^∗^	Mean PDI ± SD^∗^	Mean Zeta Potential ± SD (mV)
NLS	171.6 ± 0.8	0.152 ± 0.014	0.395 ± 0.062
SCAR1-loaded	213.0 ± 2.5	0.258 ± 0.008	0.305 ± 0.089
SCAR2-loaded	208.9 ± 2.6	0.211 ± 0.008	0.581 ± 0.021
SCAR4-loaded	185.1 ± 0.6	0.144 ± 0.012	0.318 ± 0.014
SCAR5-loaded	196.6 ± 0.8	0.178 ± 0.008	0.402 ± 0.017
SCAR7-loaded	207.8 ± 2.4	0.213 ± 0.008	0.406 ± 0.011

The PDI determines the particle size distribution, according to Kumar et al. (2015) PDI values in the range 0.01–0.5 indicate monodisperse particles, whereas PDI values > 0.7 indicate a polydisperse distribution. The PDI values of the NLS and the SCARs-loaded into the NLS were found in the range 0.144 ± 0.012 – 0.258 ± 0.008, which indicated the monodispersity of the formulations.

The zeta potential values obtained in this study were ranged from 0.305 ± 0.089 – 0.581 ± 0.021 mV, practically neutral values and according to the composition of the nanosystem, since the addition of non-ionic surfactants doesn’t affect the surface potential (Bruxel et al., 2012).

### Anti-mycobacterial and Cytotoxicity Activities

The *in vitro* results (i.e., MIC_90_, IC_50_, and SI values) for the free Ru(II) compounds and those loaded into the NLS are shown in Table 2.

**Table 2:** Results of biological assays (MIC_90_ and IC_50_) and determination of SI of the Ru(II) compounds and those loaded into the NLS against *M. tuberculosis* H_37_Rv.

Identification	Compounds	MIC_90_ - μg mL^-1^		IC_50_ - μg mL^-1^		SI – IC_50_/MIC_90_		SI
		DMSO	NLS	DMSO	NLS	DMSO	NLS	DMSO/NLS
SCAR1	[Ru(pic)(dppb)(bipy)]PF_6_	1.18 ± 0.08^a^	1.37 ± 0.07^a^	22.26 ± 1.85^a^	59.64 ± 12.28^b^	18.90^a^	43.50^b^	+130,2%
SCAR2	[Ru(pic)(dppb)(Me-bipy)]PF_6_	1.08 ± 0.12^a^	1.88 ± 0.17^b^	6.51 ± 0.67^a^	50.50 ± 1.03^b^	6.02^a^	26.86^b^	+346,2%
SCAR4	[Ru(pic)(dppb)(phen)]PF_6_	1.34 ± 0.08^a^	1.71 ± 0.17^b^	7.57 ± 0.13^a^	92.31 ± 9.06^b^	5.67^a^	53.98^b^	+852%
SCAR5	*cis*-[Ru(pic)(dppe)_2_]PF_6_	1.32 ± 0.03^a^	2.93 ± 0.12^b^	3.88 ± 0.98^a^	33.17 ± 1.23^b^	2.95^a^	11.33^b^	+284,1%
SCAR7	[Ru(pic)(dppe)(phen)]PF_6_	0.98 ± 0.05^a^	1.71 ± 0.13^b^	14.36 ± 1.57^a^	77.89 ± 8.03^b^	14.69^a^	45.56^b^	+210,2%

*For the MIC_*90*_ and IC_*50*_, the results are expressed as mean ± standard deviation (mean ± SD). MIC_*90*_, the lowest concentration resulting in 90% growth inhibition of *M. tuberculosis*. IC_*50*_, the highest concentration of compound inhibiting the growth of at least 50% of the cells. NLS, nanostructured lipid system. SI (DMSO/NLS), Ratio of selectivity indices in DMSO and NLS. Statistical analysis: Prism 7.0, one-way ANOVA – a,b: different lowercase letters on the lines indicate statistical difference by the Tukey’s test (*P* < 0.05); Equal Lyrics do not differ from each other significantly.*

Analysis of the results presented in Table 2 revealed that the anti-*M. tuberculosis* activity of the Ru (II) heteroleptic complexes was practically maintained for all incorporated compounds. However, the activity (MIC) statistically better was observed for the SCAR2-loaded, SCAR4-loaded, SCAR5-loaded and SCAR7-loaded complexes into the NLS than for those solubilized in DMSO. While comparing the activity of SCAR1 solubilized in DMSO and loaded into the NLS, no statistical difference was observed. Furthermore, a reduction in toxicity was observed in assays using macrophages for all complexes loaded into the NLS, these results showed that these formulations are statistically less toxic than the complexes solubilized in DMSO. The toxicity of some compounds was found to be reduced by 10-fold, as demonstrated by the values shown under the SI (DMSO/NLS) column; we performed a percentage comparison of the increase in the SI of the nanostructured compounds with that of the compounds solubilized in DMSO. SIs of the complexes loaded into the NLS are statistically better than the SIs of the complexes solubilized in DMSO. It should be noted that the NLS did not show any anti-TB activity. The MIC of the complexes loaded into the NLS increased only slightly. This is likely to be due to the slower release of the complexes of the matrix. In spite of this, the loaded complexes were more selective against *M. tuberculosis*, in view of the remarkable improvement in IC_50_ values. These results demonstrate that the NLS is able to reduce the cytotoxicity of Ru (II) complexes as well as maintain anti-TB activity (De Freitas et al., 2014). These results can be explained by the presence of cholesterol in the NLS. As sterol is present in greater quantities in the human body, it was chosen as a component of the system, mainly in order to reduce the toxicity of the complexes loaded into the NLS, as well as to promote interaction with the cell membrane of *M. tuberculosis*, which is bound by a phospholipid bilayer that facilitates the performance of SCAR compounds (De Freitas et al., 2014).

### Anti-mycobacterial Activity of Clinical Isolates

Table 3 shows the results of MIC_90_ determination using clinical isolates of *M. tuberculosis* from Clemente Ferreira (CF) hospital between 2007 and 2009 in São Paulo city. The strains used were 100, 158 and 169. In the determination of MIC_90_, strains of isolates CF 158 and 169 were resistant to INH and RIF and strain CF 100 showed resistance to INH, being classified with profiles monoresist and resistance MDR-TB. Table 3 also shows the genotypic resistances found by sequencing (previous work) for these strains (Miyata et al., 2011). In determining MIC_90_ of the SCARs solubilized in DMSO and loaded into the NLS, all complexes showed activity against these drug resistant strains, used in the treatment of TB. Despite the increase in MIC_90_ values, the strains tested were not resistant to the SCARs complexes, since antimicrobial resistance exhibited expressive values in relation to MIC_90_ in sensitive bacteria, as observed for the INH and RIF drugs. Of the complexes tested, SCAR4 was the only one that didn′t present statistical differences in the MIC_90_ values against the strains tested when compared to the compost solubilized in DMSO and loaded into the NLS. Regarding strain 100, only SCAR7 showed statistical differences in the value of MIC_90_, presenting a better activity of the loaded complex than the one not loaded. Also regarding the action of SCAR7 against the other resistant strains, no statistical differences were observed in MIC_90_ values. On the activity of the SCAR1, SCAR2 and SCAR5 complexes, statistical differences were demonstrated against strains 158 and 169, in which non-loaded complexes presented better MIC values than the loaded. Once the antimicrobial resistant TB indexes were declared a global threat by the WHO, new molecules that have activity against these strains, represent a great advance in the control and treatment of the disease (World Health Organization [WHO], 2017b).

**Table 3:** Panel of clinical isolates of *M. tuberculosis* with phenotypic resistance profiles determined by REMA methodology and genotypic resistance by sequencing of genes initially detected based on DNA polymorphism by PCR-SSCP (Single Strand Conformation-PCR Polymorphism).

Isolated	Phenotypic profile		Genotypic profile												MIC_90_ of the Compounds (μg mL^-1^)																	
			Isoniazid									Rifampicin																				
	REMA (MIC_90_) (μg mL^-1^)		*inhA*			*katG*			*ahpC*			*rpoB*			1-NL	1-L	2-NL	2-L	4-NL	4-L	5-NL	5-L	7-NL	7-L	AMI	CAP	GAT	INH	KAN	MOX	OFL	RIF
	INH	RIF	Nucl	Cod	aa	Nucl	Cod	aa	Nucl	Cod	aa	Nucl	Cod	aa																		
CF100	> 50	<0.195	n.d.			n.d.			n.d.			C > G	531	Ser > Trp	10.9	>12.5	5.7	3.3	5.4	2.9	5.1	5.8	7.3^∗^	3.0^∗^	0.7	5.8	0.5	>50	7.8	0.4	0.9	<0.195
CF158	> 50	> 50	n.d.			n.d.			n.d.			n.d.			2.6^∗^	11.9^∗^	1.6^∗^	5.7^∗^	2.7	3.4	2.9^∗^	5.5^∗^	3.0	2.2	0.6	1.7	0.2	>50	2.8	0.2	0.7	>50
CF169	> 50	> 50	C > T	15	Arg > Stop	T > A	337	Tyr > Asn	C > T	81	Hys > Tyr	C > T	531	Ser > Leu	2.9^∗^	12.0^∗^	2.6^∗^	11.2^∗^	3.0	5.7	2.9^∗^	8.5^∗^	4.4	3.6	0.6	1.4	1.2	> 50	1.5	1.6	11.5	>50

*Anti-TB activity of SCARs solubilized in DMSO, loaded into the NLS and of the drugs used in the therapy against clinical isolates from Clemente Ferreira Hospital CF100, which is resistant to INH, and strains CF158 and CF169, which are resistant to INH and RIF. INH, isoniazid; RIF, rifampicin; Nucl, nucleotide; Cod, codon; aa, amino acid; n.d., note detected; NL, not loaded; L, loaded. MIC_90_, the lowest concentration resulting in 90% growth inhibition of *M. tuberculosis*. Statistical analysis: Prism 7.0, one-way ANOVA with Tukey’s post-test. Significant differences between clinical isolates treated with SCARs solubilized in DMSO and loaded into the NLS are indicated by ^∗^*P* < 0.05 (Miyata et al., 2011).*

The SCAR complexes solubilized in DMSO showed promising activity against all of the resistant strains. When the SCARs were loaded into the NLS, they maintained their activity against the CF 100 strain, which is resistant to INH, as well against the 158 and 169 strains, which are resistant to both INH and RIF. The mechanism of action of these Ru (II) complexes has not yet been elucidated. Based on the results of our study, we suggest that the mechanism of action differs from that of the main drugs used in the therapy, INH and RIF.

### Intra-macrophage Activity

**FIGURE 2:**
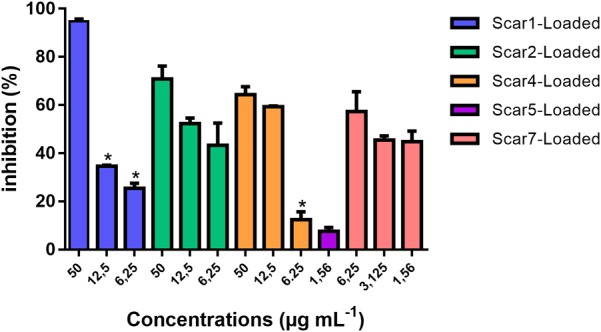
Intra-macrophage activity of Scar1-loaded, Scar2-loaded, Scar4-loaded, Scar5-loaded, and Scar7-loaded complexes after infection of J774A.1 macrophages with *M. tuberculosis* H37Rv (ATCC 27294). Statistical analysis: Prism 5.0, one-way ANOVA with Newman–Keuls post-test. Significant differences found between the concentrations of each SCAR loaded compared to its highest concentration tested are indicated by ^∗^*P* < 0.05. The percentage of inhibition was determined as the mean of three independent assays.

The results obtained from the intra-macrophagic activity assay of the SCAR compounds loaded into the NLS, in which the potential of each complex to inhibit the growth of *M. tuberculosis* within macrophages was evaluated, are shown in Figure 2.

From the results of our analysis, we can confirm that the SCARs loaded into the NLS have intra-macrophagic activity as they inhibit the growth of *M. tuberculosis* within macrophages in a concentration-dependent manner, with the exception of SCAR5-loaded into the NLS, which was evaluated only at a concentration (1.56 μg mL^-1^) due to cytotoxicity against macrophage cells in the 72-h period.

Our results demonstrate that the SCARs loaded into the NLS have intra-macrophagic activity even at MIC, with the exception of SCAR5, which in most cases does not have intra-macrophagic activity as reported in the literature, as it is much more difficult to treat intracellular bacteria due to the need to administer high therapeutic doses in order to reach the intra-macrophagic bacillus (Schwartz et al., 2006).

Although all complexes show increasing percentages of intracellular inhibition as the concentration increases, statistically this characteristic was not found for SCAR2 and SCAR7, demonstrating that even lower concentrations of these complexes are able to effectively inhibit *M. tuberculosis* within macrophages as compared to use of higher concentrations. This fact reveals a potential for SCAR2 and SCAR7 to act against intracellular bacilli more potently than the other SCARs.

For the SCAR4-loaded into the NLS the increase of the concentration of 12.5 μg mL^-1^ to 50 μg mL^-1^ also didn’t present significant difference in the intracellular inhibition potential of this complex, thus indicating that from the concentration of 12.5 μg mL^-1^ the intracellular inhibition remained constant.

### *In vivo* Oral Bioavailability

**FIGURE 3:**
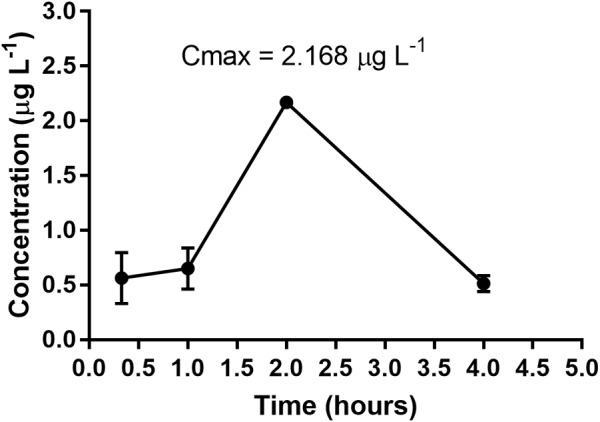
Profile of time-plasma concentration of SCAR 2-loaded NLS following a single administration via oral gavage at a dose of 300 mg/kg body weight.

The *in vivo* test of oral bioavailability involves the use of a rapid screen that reveals whether an administered complex or the final product has been absorbed into the bloodstream. Quantification via ICP-MS estimates the concentration of metal ions in the plasma in μg L^-1^ at a range of time-points following administration of the compounds in the animals by gavage; the inhibitory percentage of the complexes present in the plasma was determined using the REMA technique against *M. tuberculosis* H_37_Rv. The results of the assay confirm that the compounds are bioavailable in blood (Table 4). Figure 3 shows the time-concentration profile for SCAR 2 loaded into the NLS in plasma. For Ru quantification, Ru 102 was determined to be the most abundant and having the least interference in the analysis.

**Table 4:** Plasma levels of the SCAR complexes loaded or not loaded into the NLS following a single oral administration. For the MIC_90_ the results are expressed as mean ± standard deviation (mean ± SD) of three independent assays.

Compound	Standard	Drug Dose (mg/Kg/body)	Concentration administered (mg mL^-1^)	Mice Data							
	MIC (μg mL^-1^) Pre-determined			0.3 h		1 h		2 h		4 h	
				Inhibition (%)	ICP-MS (μg L^-j1^)	Inhibition (%)	ICP-MS (μg L^-1^)	Inhibition (%)	ICP-MS (μg L^-1^)	Inhibition (%)	ICP-MS (μg L^-1^)
Scar1-not loaded	1.18 ± 0.08	300	10.0	–	–	–	–	–	–	–	–
Scar1-loaded	1.37 ± 0.07	300	10.0	20.5	0.796 ± 0.020	18.5	8.300 ± 0.194	22.5	0.745 ± 0.034	11.0	0.606 ± 0.014
Scar2-not loaded	1.08 ± 0.12	300	10.0	–	–	–	–	–	–	–	–
Scar2-loaded	1.88 ± 0.17	300	10.0	29.0	0.562 ± 0.065	36.5	0.6515 ± 0.032	40.5	2.160 ± 0.076	31.0	0.5131 ± 0.054
Scar4-not loaded	1.34 ± 0.08	300	10.0	–	–	–	–	–	–	–	–
Scar4-loaded	1.71 ± 0.17	300	10.0	58.0	352.0 ± 0.102	28.0	15.25 ± 0.460	34.0	7.937 ± 0.122	31.0	5.247 ± 0.068
Scar5-not loaded	1.32 ± 0.03	300	10.0	–	–	–	–	–	–	–	–
Scar5-loaded	2.93 ± 0.12	300	10.0	2.00	5.672 ± 0.098	32.0	49.40 ± 0.220	22.5	14.47 ± 0.086	12.0	6.906 ± 0.095
Scar7-not loaded	0.98 ± 0.05	300	10.0	–	–	–	–	–	–	–	
Scar7-loaded	1.71 ± 0.13	300	10.0	48.0	1.455 ± 0.098	36.5	0.3577 ± 0.087	46.0	0.1598 ± 0.020	36.5	0.09344 ± 0.0031

*The percentage of inhibition and ICP-MS were determined as the mean of two independent assays.*

The calibration curves used for quantitation of plasma samples showed an *R*^2^ correlation coefficient of 0.999, and the detection limit (DL) for Ru 102 was 0.0006968 μg L^-1^. The quantification limit (QL) was 0.002322 μg L^-1^. In general, Ru 102 was detected and quantified in the plasma of the animals that received the SCAR complexes loaded into the NLS, whereas it was possible to detect the metal ion (Ru) in these animals. This result indicates that the nanostructured system has the bioavailable compounds.

The plasma concentrations for the SCAR1-loaded complex were 0.796 μg L^-1^, 8.300 μg L^-1^, 0.745 μg L^-1^, and 0.606 μg L^-1^. Plasma concentrations for the SCAR2-loaded complex were 0.562 μg L^-1^, 0.6515 μg L^-1^, 2.168 μg L^-1^, and 0.5131 μg L^-1^. Plasma concentrations for the SCAR5-loaded complex were 5.672 μg L^-1^, 49.40 μg L^-1^, 14.47 μg L^-1^, and 6.906 μg L^-1^, at 20 min, 1, 2, and 4 h following collection, respectively. The concentration of the SCAR1-loaded and SCAR5-loaded complex at 1 h was the Cmax (maximum concentration) observed i.e., 8.300 and 49.40 μg L^-1^, respectively. For the SCAR2-loaded complex, Cmax in the plasma was achieved after 2 h (2.168 μg L^-1^), after which it decayed. For the SCAR4-loaded complex, the plasma concentrations were 352.0 μg L^-1^, 15.25 μg L^-1^, 7.937 μg L^-1^, and 5.247 μg L^-1^, and for the SCAR7-loaded complex, they were 1.455 μg L^-1^, 0.3577 μg L^-1^, 0.1598 μg L^-1^, and 0.09344 μg L^-1^, for the same respective sampling times. The concentration of the SCAR 4-loaded and SCAR7-loaded complexes at 20 min was already the Cmax observed, indicating that these complexes have a faster absorption than the SCAR1-loaded, SCAR2-loaded, and SCAR5-loaded complexes.

The difference observed by comparing the time profiles of group 1 (SCAR1-loaded, SCAR2-loaded, and SCAR5-loaded complexes) and group 2 (SCAR4-loaded and SCAR7-loaded complexes) is likely to be due to their structural differences with respect to the ligands present in the coordination sphere. Group 2 has the phenanthroline ligand coordinated to the metal center and this is thought to increase the lipophilicity of these compounds compared to that of group 1 complexes, conferring a greater permeability and consequently allowing better/enhanced absorption. Segura et al. (2014) also hypothesized that greater lipophilicity could be achieved by enhancing the permeability of phen, and observed that metallic complexes containing phenanthroline coordinated to the Ag(I) ion ({[Ag(phen)(μ-tu)}_2_](NO_3_)_2_ and [{Ag(phen)(μ-tu)}_2_](CF_3_SO_3_)_2_) have a greater ability to inhibit bacteria than the complex containing bipy in the coordination sphere ([{Ag(bpy)(μ-tu)}_2_](NO_3_)_2_). This difference in inhibition was associated with the higher phen hydrophobic character, favoring a greater penetration through the membranes (Segura et al., 2014).

When comparing the compounds of Group 2 (SCAR4-loaded and SCAR7-loaded complexes), it was observed that SCAR4-loaded ([Ru(pic)(dppb)(phen)]PF_6_) was absorbed most efficiently by mice. It had a Cmax of 352.0 μg L^-1^ 20 min after administration via oral gavage, whereas the Cmax for SCAR7-loaded ([Ru(pic)(dppe)(phen)]PF_6_) was only 1.455 μg L^-1^. The significant difference in concentration may be due to the presence of an extended/elongated carbon chain in the bis(diphenylphosphino)butane (dppb) ligand present in SCAR4-loaded complex, compared to the bis (diphenylphosphino)ethane (dppe) ligand coordinated to SCAR7-loaded complex, which confers a higher lipophilicity to ([Ru(pic)(dppb)(phen)]PF_6_. Consequently, the sample had a much higher absorption. Our results demonstrate that the low surface-tension of the formulation resulted in improved solubility of SCARs-loaded complexes into the NLS and bioavailability, as compounds that were not loaded were not bioavailable (Lawrence and Rees, 2000).

In this study, we used nanotechnology methods and obtained promising results. However, the great difficulty is that there are no reports on NLS, Ru complexes, and bioavailability evaluation.

## Conclusion

The results of *in vitro* assays of SCARs loaded into the NLS demonstrate that these compounds maintained anti-mycobacterial activity against both sensitive strains and clinical isolates, compared to compounds that were not loaded into the formulation. The loaded compounds also reduced toxicity to the macrophage cell line. The SCARs loaded into the NLS showed intra-macrophagic activity in a concentration-dependent manner, with the exception of SCAR5-loaded complex. Furthermore, it was possible to determine Cmax times for the SCARs loaded in the NLS following administration via oral gavage. Our results demonstrate that the compounds become bioavailable when using nanotechnology as a tool.

## Author Contributions

PBS developed the nanostructure, analyzed the cytotoxicity, performed the *in vivo* oral bioavailability assay, quantified the Ru concentrations in plasma, and assisted in drafting the paper. EF analyzed the anti-*Mycobacterium tuberculosis* assay, performed the *in vivo* oral bioavailability assay, and assisted in drafting paper. MCS analyzed the cytotoxicity and intra-macrophagic assays and assisted in drafting the paper. PCS analyzed the cytotoxicity assay and assisted in drafting the paper. MMS and AB synthesized and characterized the complexes and assisted in drafting the paper. CE, AR, and AM quantified the Ru concentrations in plasma and assisted in drafting the paper. RC analyzed the anti-mycobacterial activity of clinical isolates and assisted in drafting the paper. MC supervised the first author (PBS), developed the nanostructure and drafting the paper. FP conceived of the project, and assisted in drafting the paper.

## Conflict of Interest Statement

The authors declare that the research was conducted in the absence of any commercial or financial relationships that could be construed as a potential conflict of interest.
