# Corrigendum: A Nanostructured Lipid System to Improve the Oral Bioavailability of Ruthenium(II) Complexes for the Treatment of Infections Caused by *Mycobacterium tuberculosis*

**DOI:** 10.3389/fmicb.2019.02517

**Published:** 2019-11-05

**Authors:** Patricia B. da Silva, Eduardo Sinésio de Freitas, Mariana Cristina Solcia, Paula Carolina de Souza, Monize Martins da Silva, Alzir Azevedo Batista, Carlos E. Eismann, Ana Marta C. M. Rolisola, Amauri A. Menegário, Rosilene Fressatti Cardoso, Marlus Chorilli, Fernando R. Pavan

**Affiliations:** ^1^School of Pharmaceutical Sciences, Universidade Estadual Paulista, Araraquara, Brazil; ^2^Department of Chemistry, Universidade Federal de São Carlos, São Carlos, Brazil; ^3^Environmental Studies Center, Universidade Estadual Paulista, Rio Claro, Brazil; ^4^Department of Clinical Analysis and Biomedicine, State University of Maringa, Maringá, Brazil

**Keywords:** ruthenium(II) complexes, tuberculosis, nanotechnology, oral bioavailability, ICP-MS

In the original article, we neglected to include the funder Grant # 2018/00163-0 São Paulo Research Foundation (FAPESP), to for all authors.

The FUNDING is: “This research was funded by grant #2013/14957-5, grant #2013/09265-7 and grant #2018/00163-0 São Paulo Research Foundation (FAPESP) by the Coordination for the Improvement of Higher Education Personnel (CAPES) together with the Post-Doctoral National Program (PNPD), the Programa de Apoio ao Desenvolvimento Científico (PADC) of School of Pharmaceutical Sciences/UNESP, Brazil and the authors thank UNESP for the data of the dissertation (Freitas, 2015).”

The authors apologize for this error and state that this does not change the scientific conclusions of the article in any way. The original article has been updated.
